# Dynamic Fuzzy-Logic Based Path Planning for Mobility-Assisted Localization in Wireless Sensor Networks

**DOI:** 10.3390/s17081904

**Published:** 2017-08-18

**Authors:** Abdullah Alomari, William Phillips, Nauman Aslam, Frank Comeau

**Affiliations:** 1 Department of Engineering Mathematics and Internetworking, Dalhousie University, Halifax, NS B3H 4R2, Canada; william.phillips@dal.ca; 2 Faculty of Computer Science and Information Technology, Albaha University, Albaha 65527, Saudi Arabia; 3 Department of Computer and Information Sciences, Northumbria University, Newcastle upon Tyne NE1 8ST, UK; nauman.aslam@northumbria.ac.uk; 4 Engineering Department, Faculty of Science, St. Francis Xavier University, Antigonis, NS B2G 2W5, Canada; fcomeau@stfx.ca

**Keywords:** wireless sensor networks, path planning, mobility models, localization models, fuzzy-logic, optimization, Fuzzy-Logic based Path Planning for mobile anchor-assisted Localization

## Abstract

Mobile anchor path planning techniques have provided as an alternative option for node localization in wireless sensor networks (WSNs). In such context, path planning is a movement pattern where a mobile anchor node’s movement is designed in order to achieve a maximum localization ratio possible with a minimum error rate. Typically, the mobility path planning is designed in advance, which is applicable when the mobile anchor has sufficient sources of energy and time. However, when the mobility movement is restricted or limited, a dynamic path planning design is needed. This paper proposes a novel distributed range-free movement mechanism for mobility-assisted localization in WSNs when the mobile anchor’s movement is limited. The designed movement is formed in real-time pattern using a fuzzy-logic approach based on the information received from the network and the nodes’ deployment. Our proposed model, Fuzzy-Logic based Path Planning for mobile anchor-assisted Localization in WSNs (FLPPL), offers superior results in several metrics including both localization accuracy and localization ratio in comparison to other similar works.

## 1. Introduction

Over the last few years, wireless sensor networks (WSNs) have been introduced as a new and simple technology for data gathering in many different physical environments [1]. A WSN typically consists of a large set of small nodes deployed in an area of interest to collect, store, and forward data and deliver it to a place where it is needed. Commonly, the nodes are low-cost, low-power, limited in terms of memory, and programmable [1,2]. WSN’s simple components and size have enabled it to be considered for many purposes in health, security, the military, and in tracking and monitoring applications [3]. In many applications of WSNs, the location of the sensor node (i.e., the location of the gathered data) is highly desired. A useful example is the location of the fire source or the exact position of pollution in some underwater applications.

Typically node deplyment in WSNs is done following an arbitrary form, where these nodes are distributed randomly especially when many nodes are used or a wide area is being monitored. Several approaches to providing nodes with their location have been proposed. An easy approach is to provide each node with a global positioning system (GPS) device. However, such solution is impractical for many reasons including the cost of the devices, the limited energy in each node and the small size of the node [4]. Another solution is to provide some nodes with their locations and let those location-aware nodes, called anchors or beacons, help the rest of the nodes in determining their own locations. These anchor nodes can be either static nodes such as those in [5,6,7], or mobile nodes such as in [8,9,10]. Mobile anchors have shown a better performance in terms of cost, coverage and accuracy [4]. The idea is to replace the many static anchors in the network with a fewer number of mobile anchors (one in many studies). The mobile anchor (MA) traverses the network to provide the nodes that are not location-aware, called unknown nodes (UNs), with their current location. Based on the planned path and the ability to receive the MA’s signals, some UNs can estimate their location. However, adopting mobility brings many challenges such as finding the path with minimum distance, the impact of the path on both the accuracy and rate of the localization, energy efficiency issues, and others.

Optimizing the travelling path of a mobile element is an active area of research in other fields including unmanned aerial vehicle (UAV) such as those in [11,12,13], robotics as in [14,15], and data collection as in [16,17]. In such areas, the mobile element starts its journey from a starting point (*a*) aiming to reach an ending point (*b*) traversing the shortest possible distance. There might be some obstacles in the area that the mobile element has to avoid. However, the objective in localization in WSN’s is different than those in UAV, robotics, or data collection. The objective is to make the mobility path as short and efficient as possible by having as many localized nodes as possible with the highest possible localization accuracy. Accuracy is represented as the localization error rate. The lower the error rate is the higher the accuracy.

In general, one of three types of movement is used in mobility-assisted localization in WSNs: random movement, static movement or dynamic movement [4]. Random mobility offers fast localization with no specific path. However, it suffers in terms of localization ratio and localization accuracy. In addition, all the UNs in the network are not guaranteed to be reached. Static mobility provides sufficient localization rate and a competitive localization error in comparison to the other types. It is an efficient solution when the MA has no constraints of movement such as limited time, limited path or limited energy. However, in scenarios where MA has a constrained movement of path length, time or energy supply, the dynamic movement is often the best candidate. The goal is to design an anchor mobility movement model, by which the MA can dynamically form its own path in a way that a maximum number of UNs are able to receive sufficient localization information to become localized. The MA path will be formed based on the real-time information from the topology of the network. The decision of each movement will be determined case-by-case. However, if the MA were to form its path based on a single input, it might affect the outcome of the model. For example, forming the movement path based on the signal strength only could result in a low localization ratio. In a similar way, forming the movement path based on the number of neighbours only might result in increasing the localization error. Thus, it is important to consider multiple criteria in order to balance the different objectives of the model.

In this paper, we present a novel dynamic Fuzzy-Logic based Path Planning for mobility-assisted Localization in WSNs (FLPPL) that applies various criteria for the movement decision to form the path of MA. The novelty of this model lies in employing multiple individual inputs in a fuzzy-logic approach for path planning that are important to minimizing the localization error and maximizing the localization ratio. By using a fuzzy-logic, an optimized movement path will be designed in order to achieve the objectives of the process and taking into account the limited movement of the mobile anchor. To the best of our knowledge, we are the first to use a fuzzy-logic based model in path planning for localization in WSNs. The proposed model offers superior results in many metrics in comparison to existing models.

Our contribution lies on designing a model for mobility-assisted localization in WSNs that:

1. For the first time, is formed based on multiple inputs. Using fuzzy-logic for processing the various inputs helps to balance the movement decision, which also helps to improve the localization ratio and the accuracy of the localization process.
2. Ensures that a maximum number of the unknown nodes in the network are able to get the localization information when the distance of movement increases, while considering the limitation of the MA movement. By doing so, a larger quantity of unknown nodes can estimate their own positions in comparison to other models.
3. Offers a competitive localization error. Implementing both the RSSI (Received Signal Strength Indicator) and the distance metrics in a fuzzy logic approach helps to improve the accuracy of the localization.
4. Uses the precision metric for a better evaluation. Precision represents how many specific localization error values are achieved as in [8]. The proposed model offers very high precision in comparison to the other existed models.

This paper is organized as follows. In Section 2, we provide an overview of related work. In Section 3, a brief review on Fuzzy-logic (FL) and some of the existing FL’s studies in WSNs. Section 4 shows the assumption for the system model. Section 5 presents our proposed model starting with an analysis of the constraints and the objectives, then shows our proposed FL system in details, and finally describe the localization process. In Section 6, we explain the simulation and performance settings, and discuss the evaluation results in Section 7. Section 8, discusses the potential extension of the current model and the future work as well as the limitations of the proposed work. Then, the conclusion part in Section 9 is provided outlining the future works as well.

## 2. Related Works

Localization in WSNs has attracted attention and several techniques and algorithms have been proposed in the recent years [4]. These techniques differ based on the objective in a number of ways such as the localization method (range-based, range-free), localization processing (distributed, centralized), deployment areas (two-dimensional, three-dimensional), application areas (outdoor, indoor), or anchor types (static, mobile).

Generally, localization models using anchor nodes are classified under one of the following types: (static nodes and static anchors), (mobile node and static anchors), (static nodes and mobile anchors) and (mobile nodes and mobile anchors) [8,18]. Our proposed model falls under the third category; hence, our related work overview will be focused in such area.

In (static nodes and mobile anchors), the movement of anchor typically follows one the three types: random, static or dynamic as described above. Based on the objective of the localization and the available sources (time, energy, etc.), a movement strategy can be chosen. Since our technique focuses on static and dynamic movement path planning, we limit our discussion only to such [8,10,18,19,20,21,22,23] techniques.

SCAN and Hilbert [18] are two of the first static mobility models proposed for node localization in WSNs. Although they open a wide area of research, they both suffer from the collinearity problem, which affect negatively both the localization ratio and accuracy. In [19], another mobility model called, CIRCLES, is formed based on circular path movement. The main goal of CIRCLES is to overcome the collinearity problem. However, this model is unable to reach the nodes located in the corners of the network, thus, affecting negatively the localization ratio. This problem can be solved by adding more outer circles to the path, however, this will bring another issue about the path length. In order to overcome the collinearity problem, Han et al., proposed a mobile anchor-assisted localization algorithm based on a regular hexagon (MAALRH) [20]. MAALRH is a static path planning technique that starts from the centre of the network and its movement path is based on a hexagon-shape movement. However, simialr to CIRCLES, MAALRH is unable to localize some of the nodes especially those around in the corners of the monitored area. Another path planning technique, called Z-Curve was proposed in [24]. The path is designed as a set of connected lines forming *Z* shapes. Another localization algorithm based on trilateration called (LMAT) is proposed in [10]. In LMAT, the movement path is designed in a set of symmetrical triangles. This approach meant to avoid the collinearity. The trilateration concept is used to increase the accuracy of localization. In comparison to other similar models such as SCAN and MAALRH, LMAT provides an excellent performance in both accuracy and ratio. In [8], a new static model is proposed. The main focus of the designed path is avoiding collinearity while guaranteeing all UNs can receive the localization information from the MA. The model succeeded to provide competitively accurate estimation with a higher accuracy in comparison to other models. A dynamic model proposes using Breadth-First (BRF) and Backtracking Greedy (BTG) algorithms to transform the path planning problem into a spanning tree [25]. However, it suffers from high error rate [26]. The dynamic path of mobile beacon (DPMB) algorithm, presented in [21], divides the nodes into three classes; unknown, settled, and reference nodes. Each node contacts its neighbours that are located in its transmission range. The MA determines its path from node to node according the weight, which is represented as a quantity of neighbours for each unknown node. Considering an exceptional scenario where the MA movement is limited or constrained, the authors in [22] suggest using a multi-hop localization technique called DV-Hop to increase the localization ratio in static path planning. With a similar assumption of the limited and constrained movement, a node localization algorithm with mobile beacon node (NLA_MB) is proposed in [23]. In this model, the area of interest is divided into a number of hexagonal grids and the MA moves from the centre of the hexagon to a corner and vice versa based on the proposed optimization model to form the final movement path. The following Table 1 provides a general comparison of different path planning schemes. The area coverage of NLA_MB differs based on the available sources of time or energy (i.e., maximum path length).

As shown in Table 1, the static models, such as CIRCLES and MAALRH, are not able to cover the whole area since the nodes located in the corner sides of the network are difficult to reach, while the accuracy is effected by the collinearity problem in SCAN and Hilbert. On the other hand, the dependency on a single parameter leads to the weak performance of coverage and accuracy in DPMB. Thus, multiple parameters in decision making of the movement direction are needed to improve the limitations of the static and dynamic models. This becomes more important in cases where the movement of the MA is constrained. The main progress of the proposed model is to overcome the collinearity problem that the static models suffer from as in [18], enable the MA to reach the any point in the network regardless of its position unlike those in [19,20], and to form the movement path based on various inputs to increase the performance of the optimization model, unlike the dynamic one in [21].

## 3. Fuzzy Logic in WSNs

Since its introduction by Zadeh in the 1960s, Fuzzy Logic (FL) has gained much attention due to its ease of implementation and simplicity [27]. The applications of FL have grown significantly. FL has been the main tool in many protocols and models in WSNs. For example, FL was used to enhance the networks lifetime as in [28,29,30]. Another usage of FL in WSNs was introduced by Mhemed et al., in [31], to propose a novel scheme, the Fuzzy Logic Cluster Formation Protocol (FLCFP), that uses Fuzzy Logic Inference System (FIS) in the cluster formation process. The work succeeded in choosing a balanced cluster head (CH) in each cluster and provides a competitive lifetime in comparison to other cluster formation models such as LEACH. Other similar works using FL are presented in [32,33,34,35]. A novel model of implementing FL to dynamically control the traffic lights using WSNs is proposed in [36]. It employs multiple fuzzy-logic controllers for a real-time traffic monitoring using WSNs. Moreover, FL was used in several data routing protocols in WSNs including those in [37,38,39,40]. However, to our knowledge, no study of using FL in path planning for mobility-assisted localization in WSNs has been proposed. Therefore, we introduce our FLPPL model.

**Figure 1:**
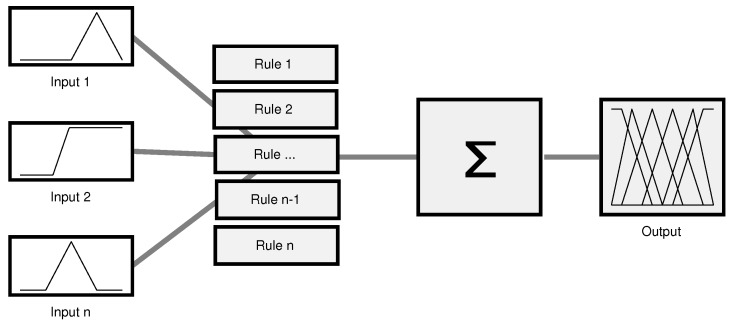
Fuzzy-logic system structure.

Simply, the concept behind it is to smooth the classical Boolean logic of True (1 or yes) and False (0 or No) to a partial value located between these values coming in a term of the degree of membership [31]. Such degree is determined based on multiple inputs, a decision function and one or more output functions. The set of inputs will be tested and evaluated together according to the applied set of rules. Rules works as a medium for the logical relationship between the inputs and the outputs. Thus, the results of the rules are combined and defuzzified in order to produce the degree of the final output [27]. Figure 1 depicts the generic structure of the Fuzzy-logic system as illustrated in [27].

## 4. System Model and Assumptions

The following features are assumed to form the system model:

1. A two-dimensional square network. The area size of the network is denoted as *S* in $m^{2}$.
2. A collection of unknown nodes, UNs, are distributed randomly around the network. The number of UNs is denoted as *N*.
3. Initially, all UNs are not location-aware.
4. The deployed nodes are stationary, thus, no change to their location after deployment.
5. Each sensor node has a stable communication range of $R_{Tx}$ in *m*.
6. A mobile anchor, MA, can determine its own position at any point of the network area. It is able to travel freely around the entire network in straight directions. The number of MAs is denoted as *M*.
7. For simplicity, no obstacles in the deployment area are considered.
8. The movement distance of the MA is limited by the value of the maximum distance to travel, $d_{max}$, where the MA’s movement cannot exceed that value.
9. The MA stops frequently to provide nodes with information containing its current position and continue moving. Each stopping point is called a localization point.
10. Each MA and UN can contact each other only if their locations are within the communication range $R_{Tx}$.
11. Once a UN receives any three different locations information, it will be able to estimate its own location using the applied localization algorithm.
12. Each node that succeeds in estimating its location, will be converted from a UN to a reference node, RN. Each RN can share its location with the other nodes, helping them to estimate their own locations.

## 5. Proposed Model

### 5.1. Constraints and Objectives Analysis

The main objective of the designed model is minimizing the localization error while maximizing the localization ratio. Similar to most path planning models, a number of constraints are considered in planning the MA movement as follows. First, every visited localization point is unique; this means the MA cannot visit a localization point more than once. Second, in order to avoid the collinearity of localization points, the optimization requires that every three consecutive localization points are not collinear. In addition, and as in [22,23], another constraint is considered in designing this model that the movement distance is limited, which therefore means MA cannot exceed the maximum traveling distance. These constraints may be broken in only one case where the MA trapped in a corner or a border of the network. Two objective functions are to be optimized, the minimum average localization error and the maximum localization ratio. They are respectively represented in the following formulas

(1) $$Minimize\;Error_{avg}$$

(2) MaximizeRN

### 5.2. Fuzzy-Logic Based Movement Decision

**Figure 2:**
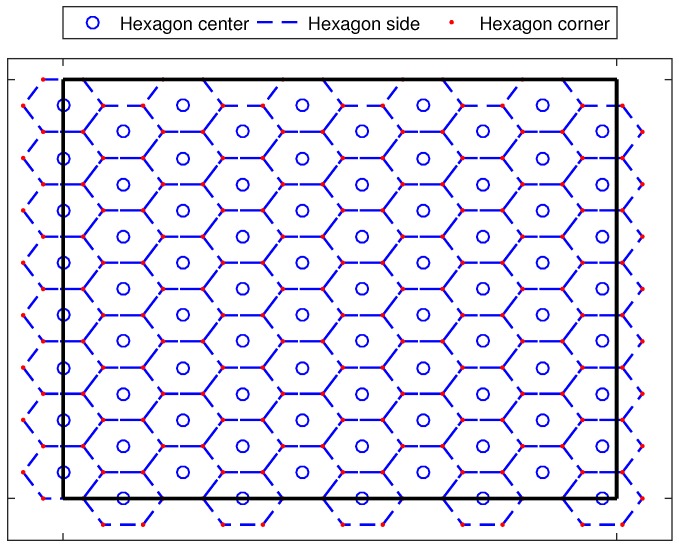
The hexagonal network system model of FLPPL (Fuzzy-Logic based Path Planning for mobility-assisted Localization).

The first step in path planning starts with dividing the area of interest into a set of symmetric virtual hexagons. The MA is supposed to travel from the center of one hexagon to one corner of the same hexagon and the reverse procedure is applied in the next movement. Hexagonal-based movement has been proposed in a number of recent works including [21,23,41]. Similar to [23], the hexagons are formed based on the transmission range of $R_{Tx}$, where the side length of each hexagon is equal to $R_{Tx}/\sqrt{3}$ *m*. Figure 2 shows an example of the virtual hexagons forms in our system.

The movement of MA from one point (either the center or a corner) to another point will be determined in a real-time approach using an FL system. In such system, three inputs will be used namely the Received Signal Strength Indicator (RSSI) level, the number of neighbours, and the distance to each neighbour. These inputs will be measured from the MA’s current location. The Mamdani method was used as the main tool to represent the fuzzy system. Mamdani is considered to be intuitive, widespread accepted and well suited to human input [27]. Table 2 shows the three input functions along with their membership functions.

RSSI input measures the strength of the signal of all unknown nodes located within the range of the current location of MA. A scale of −100 dBm to 0 dBm is used in this model, where the closer the value to 0, the stronger the signal is [42]. The RSSI membership function used in this model is close to the one in [43]. All UNs within the range of the MA will exchange their neighbour table information with the MA. This table includes the node id, the number of neighbours, the neighbour ids, the RSSI strength and the localization status, whether localized (1) or not (0). An example of this table is shown in the following Table 3.

In the second input, and from its current location, the MA contacts each UN located within its range. Each UN exchanges its neighbours list and the MA evaluates them based on the number of neighbours. A higher chance will be given to the UN with higher number of neighbours. This is meant to reach the maximum number of UNs by considering the ability of each nodes to share its information to as much neighbours as possible. In the third input, although the locations of both the MA and the UN are required to calculate the distance between each of them, it is still possible to estimate using the RSSI. The closer the distance is, the higher chance is given. Considering the module in [44] and assuming $d_{0}$ = 1, the distance extracted from the RSSI values is estimated as

(3) $$d=10^{\left(-RSSI+P_{L}\left(d_{0}\right)+N_{\alpha }\right)/\left(10\times \beta \right)}$$

where $P_{L}\left(d_{0}\right)$ is the power loss at the reference point $\left(d_{0}\right)$ in dB, $N_{\alpha }$ is the zero-mean Gaussian random variable with a standard deviation α and β is a constant path loss exponent.

Two membership functions were chosen to present our parameters, Triangular and Trapezoidal. They are simple, direct and easy to apply in many applications [27,45]. Simply, triangular membership function is a collection of three points forming a triangle. It is represented using the following equation

(4) $$\mu_{A1}\left(x\right)=\{\begin{array}{ll} 0 & x\leq 0\\ \frac{x-a1}{b1-a1} & a1\leq x\leq b1\\ \frac{c1-x}{c1-b1} & b1\leq x\leq c1\\ 0 & c1\leq x \end{array}$$

Trapezoidal membership function is like the Triangular function with a flat top. It is formed as

(5) $$\mu_{A2}\left(x\right)=\{\begin{array}{ll} 0 & x\leq a2\\ \frac{x-a2}{b2-a2} & a2\leq x\leq b2\\ 1 & b2\leq x\leq c2\\ \frac{d2-x}{d2-c2} & c2\leq x\leq d2\\ 0 & d2\leq x \end{array}$$

As in [31,32], we used the triangular function for the middle variables while the trapezoidal member function was used for the boundary variables. The values used in both the Triangular and the Trapezoidal membership functions are shown in the following Table 4.

**Figure 3:**
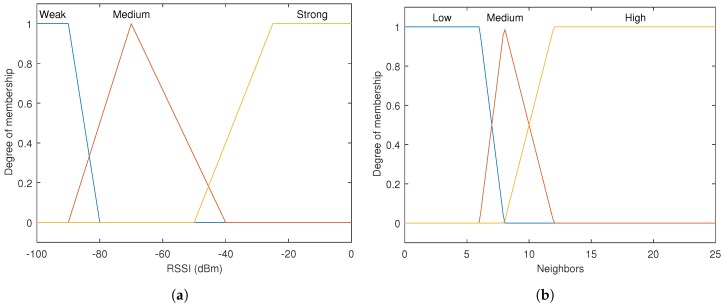

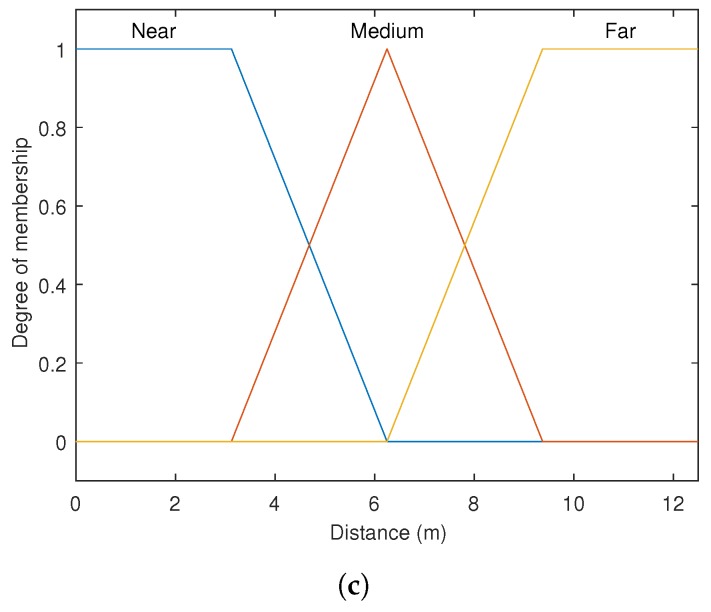
Inputs functions and degrees of relationship of FLPPL, (**a**) the RSSI level input, (**b**) the number of neighbours input and (**c**) the distance to each neighbour input.

Figure 3 illustrates the inputs functions and degrees of relationship of FLPPL, Figure 3a the RSSI level input, Figure 3b the number of neighbours input and Figure 3c the distance to each neighbour input.

**Figure 4:**
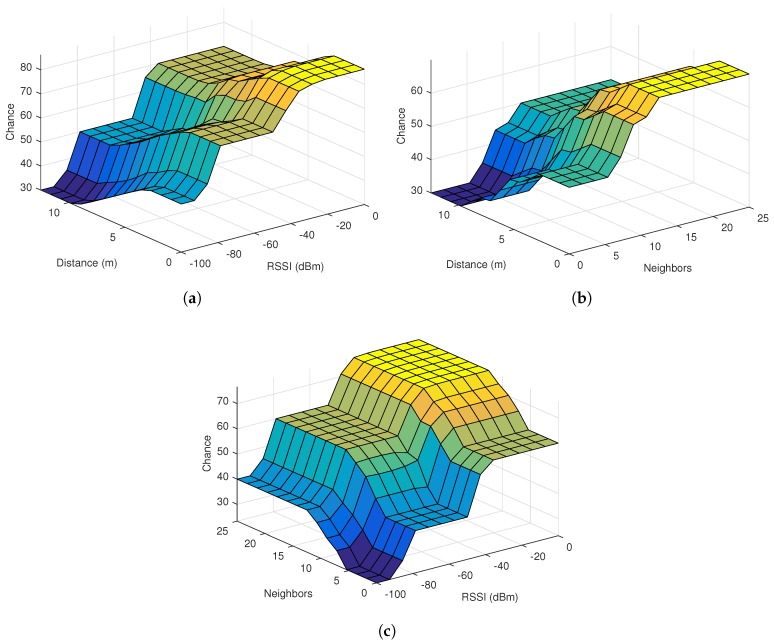
Relationship among fuzzy inputs of FLPPL, (**a**) distance vs. RSSI, (**b**) Distance vs. Number of Neighbours, (**c**) Number of Neighbours vs. RSSI. The darker the color is, the lower the chance is.

The correlations between each two input functions and the output can be represented as a three-dimensional curve. The following surface plots in Figure 4, depict the correlations between the fuzzy inputs in our FLPPL approach, starting with the Distance to RSSI as in Figure 4a, Distance to Neighbours in Figure 4b and Neighbours to RSSI in Figure 4c.

**Figure 5:**
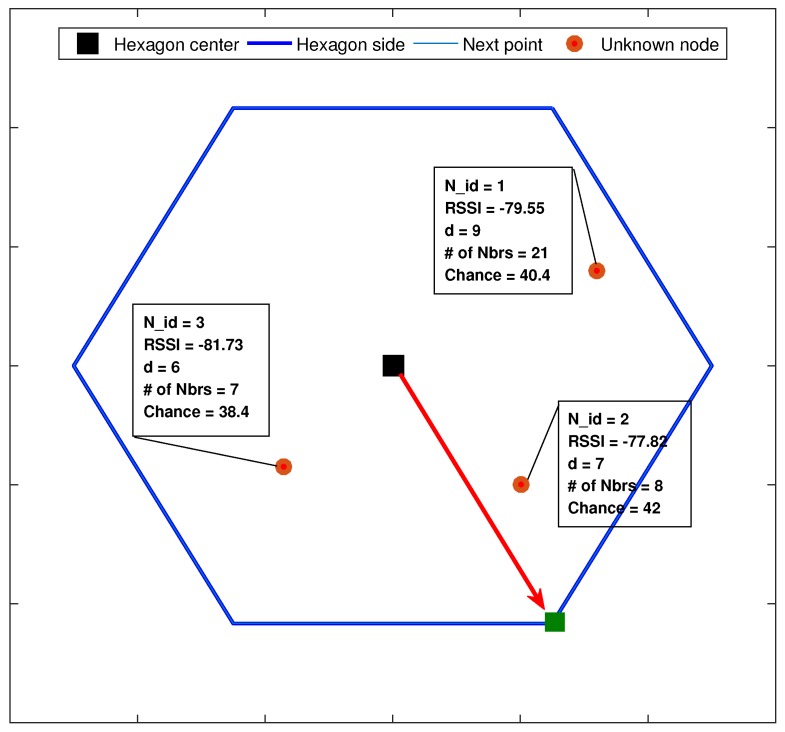
Example of selecting the next movement of the MA.

The system output function in our model is identified as the chance (probability). The chance represents the probability of the MA’s next point from the current location. The MA will move to the closest point in distance to the higher chance node. For example, consider the three nodes (N_ids = 1, 2 and 3) shown in Figure 5. The MA current location is in the center of the hexagon, which by default means that the next movement will be to a corner of the hexagon. Now, the MA will contact each node, exchange their information and analyze it. Using the fuzzy-logic model, the MA will estimate each node’s chance considering the three membership function inputs. While still taking into account the rules of constraints described in Section 5.1, the MA will move to the closest point (in green) to the highest chance node (N_id = 2 in the example).

Nine output degrees are defined in this system similar to those used in [31]. They range from (very weak) as the lowest degree to (very strong) as the highest one. Table 5 shows the output function along with the nine membership functions.

Any fuzzy-logic system depends on the IF-Then rule statements that are used to formulate the conversion from the input functions to the output one. A simple fuzzy IF-Then rule is stated a

(6) ifxisA,thenyisB

where *A* and *B* are the linguistic values defined by the fuzzy sets on the ranges *x* and *y* [27]. The following Table 6 defines the fuzzy if-then rules.

**Figure 6:**
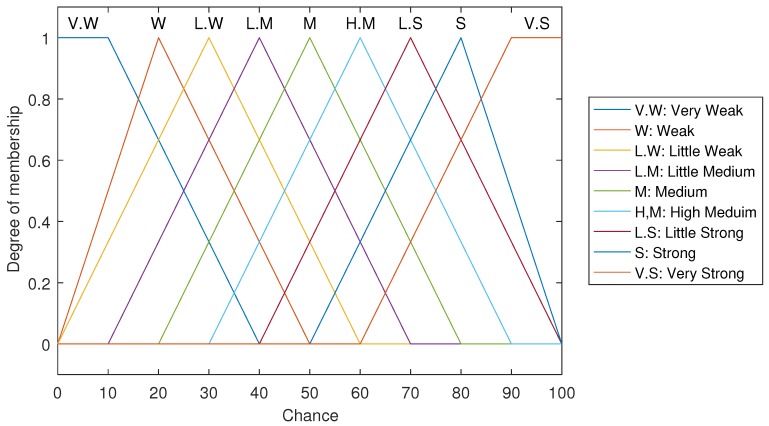
Output membership function of FLPPL.

Figure 6 plots the degree of Membership, in the range of 0 to 1, versus the chance values of FLPPL.

**Figure 7:**
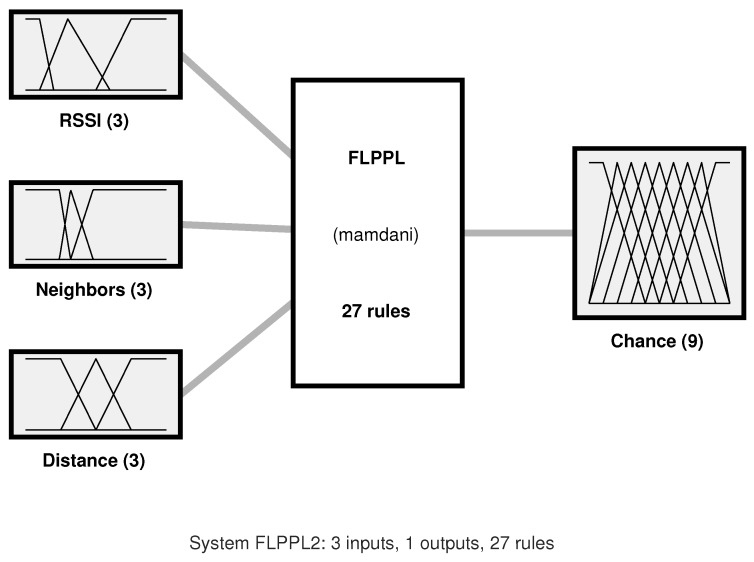
The system scheme of FLPPL.

Therefore, the complete system model of FLPPL consists of three inputs with three membership functions and nine degrees of chance as shown in Figure 7.

### 5.3. Mobility Movement and Localization Process

The mobility movement and localization process are conducted simultaneously in two sides, the UN’s side and the MA’s side as follows.

#### 5.3.1. Procedure in Unknown Node’s Side

1. UNs will be deployed randomly.
2. Each node will communicate with its neighbours’ node that are located within its communication range, collecting their information and adding them to its neighbours table was shown above in Table 3.
3. When MA arrives at each node, the node will exchange its table with the MA.
4. When three different locations are received by each UN, it will be able to calculate its own location.

**Figure 8:**
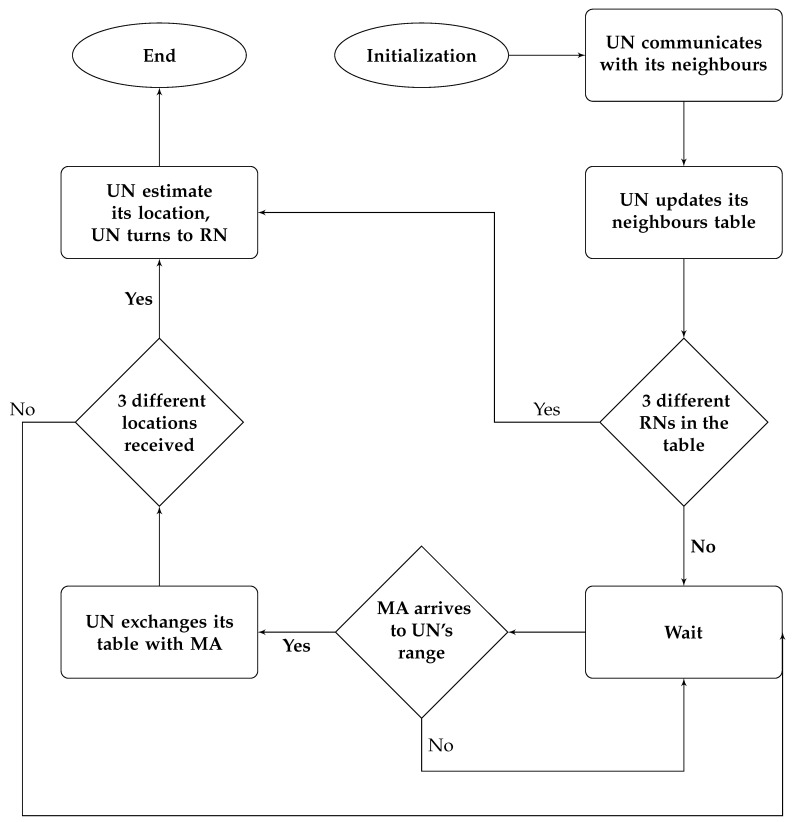
The node localization process in the UN.

The flowchart in Figure 8 presents the localization procedure done on the UN’s side.

#### 5.3.2. Procedure on MA Side

1. MA will start its journey from a starting point, the starting point can be set in advanced or random.
2. MA has a maximum distance value.
3. The first three movements will be random in any direction.
4. After each movement, the MA will stop and communicate with all nodes in its communications range, providing them with its current position.
5. It will update its routing table, which has the following information:
  (a) Node IDs
  (b) Node’s status
  (c) Number of neighbours
  (d) Neighbours IDs
  (e) Neighbours’ status
  (f) RSSI value
6. The MA will evaluate all nodes and elect one based on the previous chance table.
7. The next point of the hexagonal will be the shortest point in distance to the elected node.
8. MA will travel to that point and provide its current position information.
9. MA keeps moving till reaching the maximum distance, $d_{max}$.

**Figure 9:**
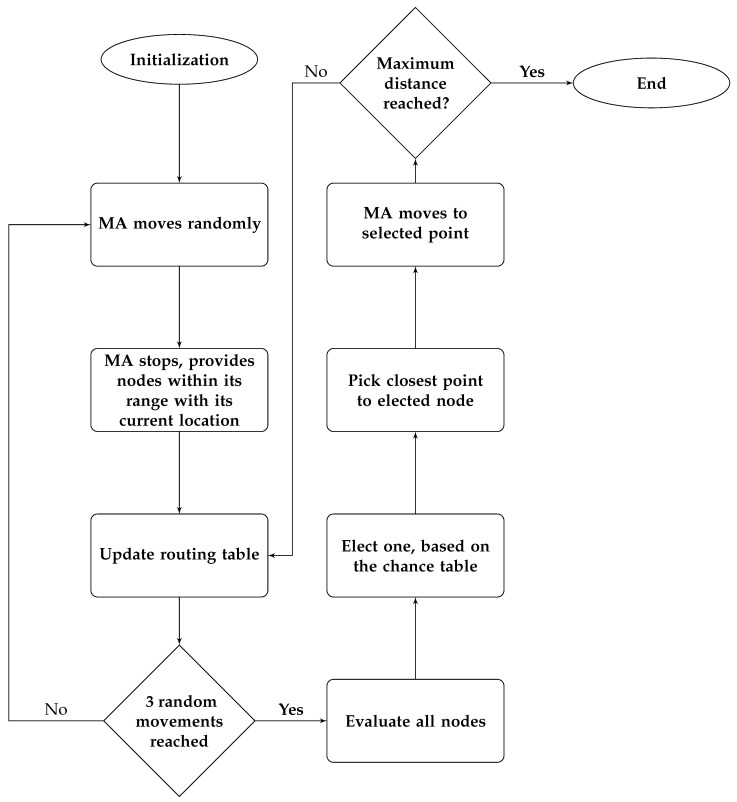
The movement and node localization process in the MA.

The flowchart in Figure 9 presents the localization procedure done in the UN’s side.

## 6. Performance Settings

Three static path planning algorithms and one dynamic model were implemented to evaluate the performance of FLPPL. SCAN, LMAT and Z-Curves are the static models while the NLA_MB model was used as a dynamic algorithm.

**Figure 10:**
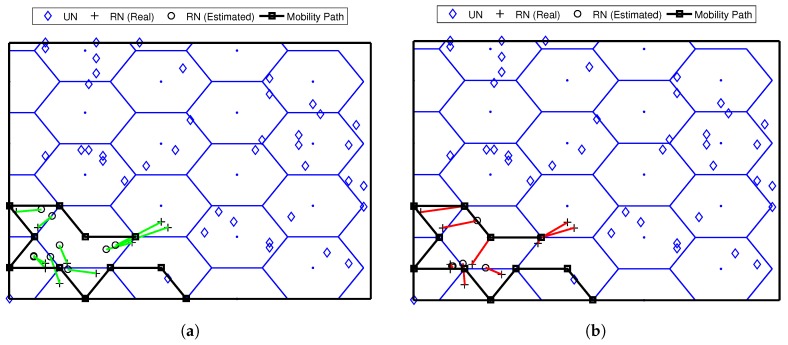
The FLPPL mobility movement and the estimation of location of the same nodes deployment in (**a**) WCL, and (**b**) WCWCL.

Two localization algorithms, Weighted Centroid Localization (WCL) [46] and Weight-Compensated Weighted Centroid Localization (WCWCL) [44], were implemented to analyze the effectiveness of the mobility models implemented. WCL is a simple localization algorithm that provide a localization estimation with a relatively low communication consumption and a lower energy cost. The location estimation is based on the weights of the anchors and their estimated distance. WCL has been used as the main tool of localization in several path planning models. WCWCL is an extended and improved version of WCL. It gives more impact for the nearby anchor nodes to calculate the weights which therefore provides more accurate estimation. Figure 10 shows an enlarged example of the FLPPL mobility movement and the estimation of location of the same nodes deployment in the two algorithms.

We evaluated the performance of the proposed FLPPL model using MATLAB environment and compared it with the other implemented models of SCAN, LMAT, Z_Curve and NLA_MB in terms of localization accuracy, localization precision and localization ratio and coverage respectively. The simulation tool and implemented parameters were chosen in accordance with some other similar works, we used them here for consistency. A square network area is assumed with a size, *S*, of 100 m × 100 m. A randomly distributed set of 250 nodes, *N*, is used with only one mobile anchor, *M*. The maximum movement, $d_{max}$ indicates the maximum distance that the mobile anchor can take in travelling around the network. Different maximum movements were used to evaluate the models. A random starting point approach was used in each run for each model. A realistic wireless model was used by implementing the characteristics of a wireless node that is equipped with a Chipcon CC1100 radio module [47]. Such specification were already used in similar works including [8,44].

The rest of the parameters are shown in the following Table 7.

## 7. Evaluation and Results

To assess the efficiency of the proposed model, we studied the models considering the following aspects, discussed below: accuracy, precision, and localization ratio.

### 7.1. Localization Accuracy

Localization accuracy is one of the main criteria of evaluation when designing a mobility path planning. The higher the accuracy is, the more successful the designed model. Thus, it is considered as the prime factor in many works. Accuracy is defined based on the achieved localization error. The lower the localization error is, the higher the accuracy is. In this work, we study the accuracy from two perspectives, the average localization error and the standard deviation of the localization error in each model.

#### 7.1.1. Average Localization Error

The average localization error is indicated as the calculated distance between the real location and the estimated location of the localized node. It is used to determine the degree to which the localization estimation is accurate. The node localization error is formulated as

(7) $$error_{\left(i\right)}=\sqrt{{\left(x_{i}-u_{i}\right)}^{2}+{\left(y_{i}-v_{i}\right)}^{2}}$$

where $\left(x_{i},y_{i}\right)$ are the true coordinates of the node *i*, and $\left(u_{i},v_{i}\right)$ are the estimated ones of the same node *i*. Therefore, the average localization error, $error_{avg}$, for the entire number of unknown nodes, *N*, is calculated as:

(8) $$error_{avg}=(\sum_{i=1}^{N}error_{\left(i\right)})/N$$

where *N* is the total number of sensor nodes.

**Figure 11:**
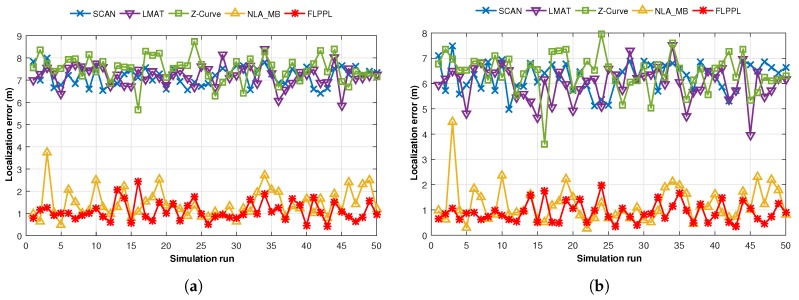
Localization errors of all mobility models in (**a**) WCL, and (**b**) WCWCL, ($d_{max}=70,\sigma =3$).

However, to study the behaviour of the proposed model and compare it to the others, we first executed a test of 50 simulation runs with 250 unknown nodes, a maximum movement distance, $d_{max}$, of 70 m, and a standard deviation of noise, σ, of 3. Figure 11 shows the performance of the different models based on localization error when both WCL and WCWCL were used.

Figure 11a shows that FLPPL offers lower error rate, thus, higher localization accuracy in most of the presented results when WCL was used. A range of localization errors between 0.434 m and 2.439 m were achieved. The dynamic path planning of NLA_MB also presented a better performance in comparison to the other static models of SCAN, LMAT and Z-curves. While LMAT was proposed for a better performance as a static model [8,10], when the MA movement is constrained, there is no significant difference to the other static models. This may help to prove that the dynamic path planning models are more suitable in such cases where the MA movement is limited and there is a need to take the network topology and nodes deployment into account in the movement decision making.

The same assumptions were used again with WCWCL scheme, as shown in Figure 11b. Higher localization estimation was achieved by decreasing the localization error using WCWCL. FLPPL again provided higher performance of accuracy by offering lower localization error, which therefore means more accurate estimation. Once more, NLA_MB shows higher estimation of node locations with minimal locations error compared to the implemented static models. On average, WCWCL attained better results than WCL due to the fact of given the nearby anchors more impact on the estimation and calculation of the locations. For example, FLPPL yielded an average of 0.898 m with a range of errors between 0.345 m and 1.960 m in comparison to the higher results of the same assumptions in WCL.

**Figure 12:**
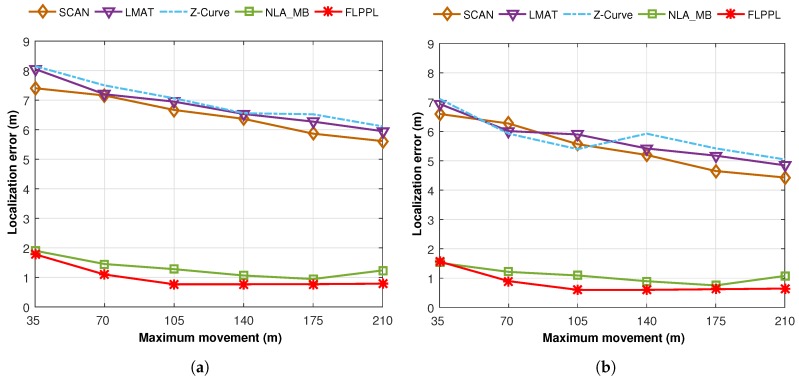
Average localization errors versus maximum movement in (**a**) WCL, and (**b**) WCWCL.

Moving on to the average localization error for all path models when different maximum movement distance, $d_{max}$, are used, Figure 12 shows the performances when WCL and WCWCL are implemented. We conducted two experiments using the parameters shown in Table 7 with two changeable values, the maximum movement distance, $d_{max}$, and the standard deviation of noise, σ.

In the first trial, we ran a test of 250 unknown nodes with different movement distances ranging between 35 m and 210 m and σ=3.

Figure 12a presents the average localization error for the proposed model along with the others when WCL is used, while Figure 12b shows the average localization error when WCWCL is used. FLPPL and NLA_MB offered the best results with slight distinctions in both WCL and WCWCL. FLPPL shows superior results with all movement allowances. In general, WCWCL provides better results with all path models including the statics. However, the difference between the dynamic models of FLPPL and NLA_MB, and the rest of SCAN, LMAT and Z-Curve is very significant. For example, the localization error of the models when only 35 m of distance is allowed are 7.41, 8.06, 8.15, 1.90 and 1.78 m for SCAN, LMAT, Z-Curve, NLA_MB and FLPPL respectively when WCL is used. These results improved to 6.59, 6.94, 7.11, 1.52 and 1.56 m when WCWCL is used.

Two interesting points here to mention. First, while increasing the movement distance showed better localization in most trials, this not always true. Increasing the maximum movement distance does not always mean that the accuracy will increase as shown in the figures. Second, the static models behave differently based on the implemented localization algorithm. For example, Z-Curve shows better performance than SCAN and LMAT when WCWCL is used with 105 m.

**Figure 13:**
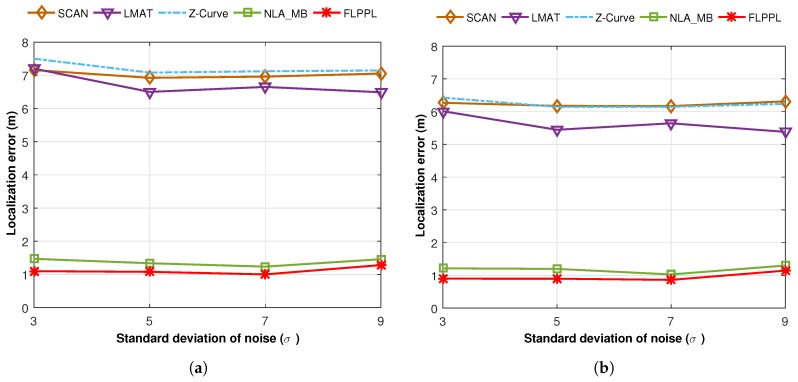
Average localization error versus the standard deviation of noise (σ) of the mobility models in (**a**) WCL, and (**b**) WCWCL.

In the second experiment, shown in Figure 13, we conducted the same parameters with a fixed maximum distance, $d_{max}$, of 70 m and a different standard deviation of noise, σ.

Figure 13a,b shows the average localization error for the chosen models with a different deviation od noise, σ, when WCL and WCWCL algorithms respectively are used. In all runs and regardless of the value of the standard deviation of noise, σ, our proposed model, FLPPL, offered the most accurate estimations, with a localization error of less than 1.5 m in both WCL and WCWCL. Indeed, FLPPL was able to achieve even lower results than 1 m when the values of 5 and 7 were used as standard deviation of noise, σ in WCWCL. NLA_MB showed closed results and the impact of changing the standard deviation of noise, σ, was insignificant. Static models offered better results when σ increases to 5. Once more, static models with WCWCL outperformed those with WCL.

To sum up this point, taking into account the strength of the node’s RSSI signals, locations and distance of both the MA localization points and the localized neighbour nodes helped to achieve high accuracy values in FLPPL using both WCL and WCWCL. Moreover, in FLPPL, the localized nodes are given the opportunities to provide their neighbours with their own localization information, therefore, the unknown node can determine its own location based on the most accurate information received. NLA_MB also consider the usage of the nearby localized nodes to supply their own neighbour with their locations, which helps to increase such ratio.

#### 7.1.2. Standard Deviation of the Localization Error

**Figure 14:**
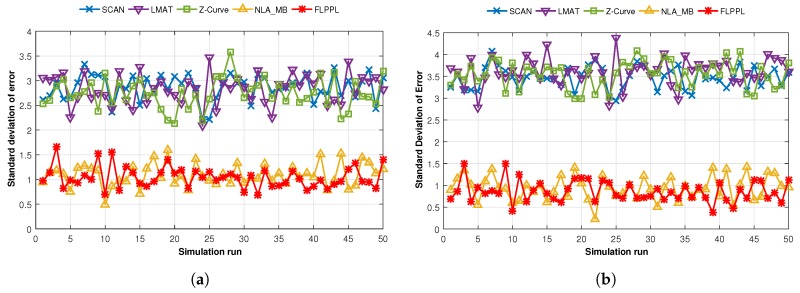
Standard deviation of errors of all mobility models in (**a**) WCL, and (**b**) WCWCL.

In addition to low localization error, a low standard deviation in localization error is desirable. The standard deviation of localization error is formulated as

(9) $$error_{std}=\sqrt{\frac{1}{N}\sum_{i=1}^{N}{\left(error_{\left(i\right)}-error_{avg}\right)}^{2}}$$

where *N* is the UNs number, error(i) is the localization error for each node *i*, and $error_{avg}$ is the average localization error. We examined the standard deviation of error from two aspects as in past experiments, the change of maximum movement distance, $d_{max}$, and the change of standard deviation of noise, σ. Figure 14 depicts the standard deviation of localization error rate for WCL and WCWCL, when $d_{max}$ = 70 and σ = 3 for each run time.

When WCL is used, as shown in Figure 14a, both FLPPL and NLA_MB interchangeably present low standard deviation of error results ranging between 0.68 m and 1.66 m in FLPPL, and 0.49 m and 1.59 m in NLA_MB. However, this is not the case when SCAN, LMAT or Z-Curve are used. Higher standard deviations of error are offered in all the 50 runs. One reason behind that is the nature of the design of the static models that does not consider the requirements of the network and the movement constraints. Even in the other scenario, where WCWCL was used, FLPPL and NLA_MB provided very small deviations with minimum values that could reach 0.38 m in FLPPL and 0.22 m in NLA_MB. Figure 14b shows the results of the all movement paths behaviour in WCWCL over the 50 simulation runs. However, while WCWCL improved the standard deviation of error results in both dynamic models, it increases the deviations in all the static models.

**Figure 15:**
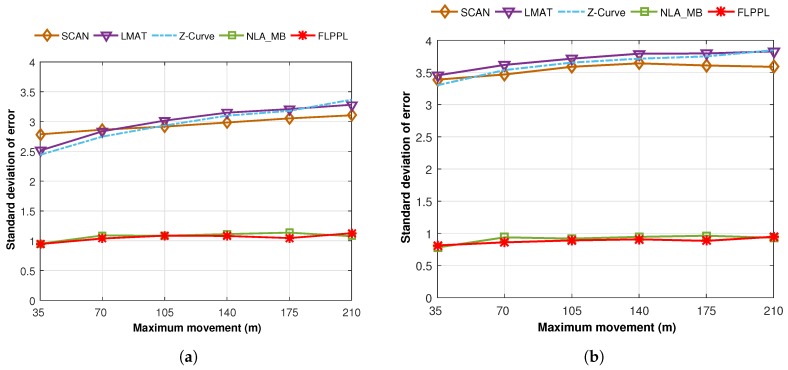
Standard deviation of errors versus maximum movement in (**a**) WCL, and (**b**) WCWCL, ($d_{max}=70,\sigma =3$).

We also calculated the average standard deviation of error of 50 simulation runs using different maximum distances, $d_{max}$ which are shown in Figure 15.

In Figure 15a, the average standard deviations of error are shown when different maximum distances, $d_{max}$ of 35 m, 70 m, 105 m, 140 m, 175 m and 210 m are used with WCL and σ is 3. Broadly, increasing the maximum movements increases the standard deviation of error especially when the static models are used. LMAT and Z-Curve show better results than SCAN when 35 m is used, however, the results change gradually till SCAN surpassed the other two when 210 m of movement is used. Both dynamic models kept the deviations around 1 m regardless of the value of $d_{max}$. When WCWCL is applied, as shown in Figure 15b, the dynamic models again outperformed the other models with lower deviations this time with less than 1 m in all run times. However, the deviations increased in the static models when using WCWCL compared to WCL. Still, the deviations increase when the movement increases in all models.

### 7.2. Precision

**Figure 16:**
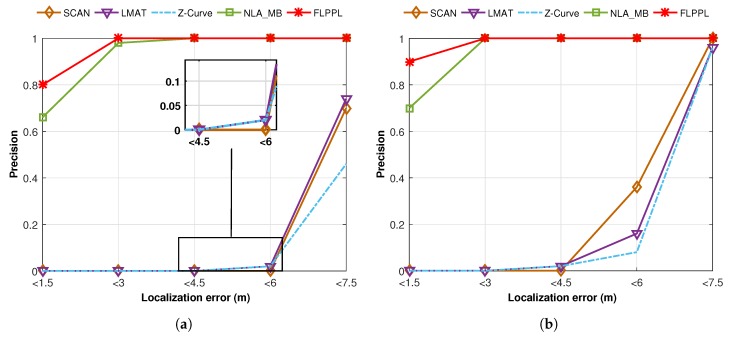
Precision of all mobility models versus the localization error in (**a**) WCL, and (**b**) WCWCL, ($d_{max}=70,\sigma =3$).

For evaluation in more than one criteria, we also used the precision metric, which represents the degree of how many particular accuracy values are reached [3,8]. Five localization error values are considered as follow, <1.5 m, <3 m, <4.5 m, <6 m and <7.5 m. The ratio of how many values were attained is calculated with each of the previous localization error values in the 50 simulation runs. Figure 16 shows the precision evaluation results when $d_{max}$ = 70 and σ = 3.

In Figure 16a and when WCL is used, the number of the localized nodes with localization error within 1.5 m is very high with over 0.8 precision in FLPPL. Again, the reason behind it is the consideration of employing both the RSSI values and the distance metrics in the decision movement which therefore leads to a better localization. In fact, all localization error values were kept in control under 3 m in FLPPL. NLA_MB also provided a high precision when compared to the other static models with more than 0.6 within less than 1.5 m and more than 0.9 precision within less than 3 m of errors. The static models of SCAN, LMAT and Z-Curve acted very weakly only few values reached within 6 m of the real locations. This precision increased slightly with 0.46 in Z-Curve, 0.7 in SCAN and 0.74 in LMAT when a localization error of 7.5 m is considered which means the rest of the localized nodes in each model were achieved in more than 7.5 m or error rate.

When WCWCL is applied, the precision was improved as shown in Figure 16b. This is because WCWCL modified the weight calculation and gave more effect of the distance and nearby anchors. FLPPL obtained about 0.9 of precision where localization error was kept less than 1.5 m. All FLPPL localization errors were under 3 m of error. Even in NLA_MB, the precision increased from 0.66 when WCL was used to 0.7 in WCWCL when less than 1.5 m was considered. The impact of WCWCL is clear on the static models precision as well. The precision values improved gradually from 4.5 m to less than 7.5 m.

### 7.3. Localization Ratio

Localization ratio represents the quantity of localized nodes divided by the total number of UNs. It also indicates the coverage of localization that MA can make. The higher the localization ratio is, the more successful the model. The localization ratio is given as

(10) $$L_{avg}=\frac{RN}{N}$$

where RN is the total number of successfully localized nodes (reference nodes) and *N* is the total number of the original distributed unknown nodes.

**Figure 17:**
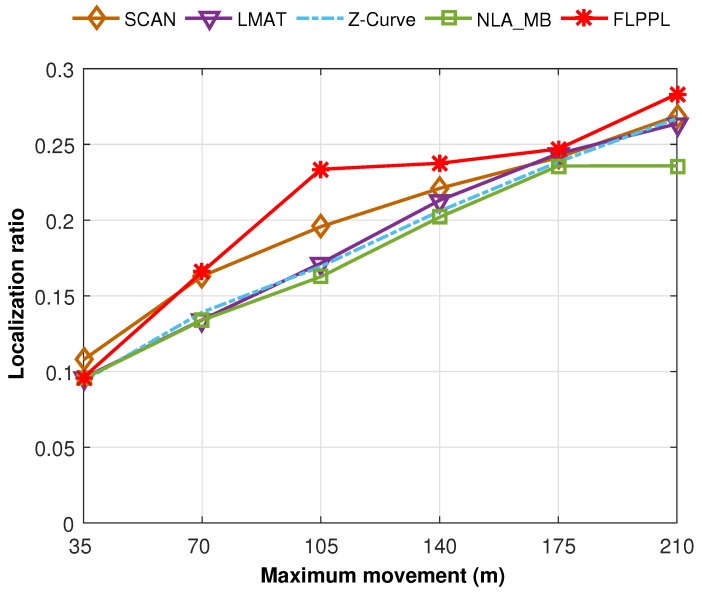
Localization ratio all mobility models in both WCL and WCWCL.

A set of 250 unknown nodes is used with different movement distances, $d_{max}$ with the default value of standard deviation of noise, σ = 3. Since the localization ratio is almost the same in both WCL and WCWCL, we only show one of them here. Figure 17 presents the localization ratio for the implemented path models.

When $d_{max}$ = 35, all paths, including FLPPL and NLA_MB, provided insufficient ratio of localization. The very short movement allowance had its negative impact on the number of localized nodes. With increasing the maximum distance, the localization ratio improves. FLPPL is the only model that takes the number of neighbours of each node in its movement decision. Hence, it showed a superior performance starting with $d_{max}$ = 70 till $d_{max}$ = 210. Another reason is that nodes in FLPPL are able to share their own location, which helps to spread the localization information. This feature is applied in NLA_MB as well. However, while NLA_MB provides a competitive localization error, the other static models offered higher localization ratio in most cases. The distance between each two points in the static models is farther than those in NLA_MB which means more nodes will receive the localization information than those in NLA_MB.

## 8. Discussion

In this section, we discuss the possible extension of the proposed model for our future work. We also highlight the limitations and the points that are not considered in this work.

### 8.1. Extension and Future Work

In this work, we assumed that the area of interest is obstacle-free. Therefore, we did not consider the ability of model to work when obstacles exist nor how the MA can avoid them. As a future work, we aim to study the effects of the obstacles existed in the area on localization accuracy, localization ratio, precision and the path length. Moreover, we will work on proposing a technique for the MA to sense and avoid the obstacles and continues its journey. We also plan to extend our experiments to the three-dimensional scenarios and test its ability to work in such environments. Another task is to assess the current model when multiple MAs are used. Recently, a new research area suggested using the Particle Swarm Optimization (PSO) to optimize the membership functions of the Fuzzy Logic Controller (FLC) in WSNs [48]. PSO has shown promising results in many research including [49,50]. Such proposal can be useful to improve the performance of our model.

### 8.2. Limitations

The proposed model was designed for a densely deployed network, assuming that the area includes a sufficient amount of sensor nodes. In case there is an insignificant number of sensor nodes, the inputs of the fuzzy system might be affected (especially the number of neighbours), thus, affects the optimization of the path planning. On the other hand, most of the model operations exist in the MA’s side. Assuming that the MA is not energy-constrained and is able to complete the assigned movement distance, we did not consider the computation load or energy consumption in this model. In a special scenario where the MA has a trivial source of energy, it might be unable to even complete its limited distance. Although our proposed model shows a significant improvement of localization ratio in comparison to other models, it is very difficult to guarantee that all UNs will be able to receive the localization information. Two reasons behind that namely the limited movement distance of the MA, and the nature of the dynamic models of forming the mobility movement based on the information received from the deployement area.

## 9. Conclusions

In this paper, we introduced a dynamic mobility path model for node localization in WSNs. The proposed path model is formed based on a number of inputs in a fuzzy-logic approach which helps to design a superior optimized path when the movement of the MA is limited. Two types of mobility, represented by four different models, are implemented in this work to test and examine our proposed model. The results show that our model, FLPPL, increases the network’s localization efficiency in terms of localization coverage, localization accuracy, and localization precision. We were able to draw conclusions based on the three metrics studied:

1. **Localization accuracy:** Represented by the localization error, the final results show leading outcome for our proposed model along with the dynamic model of NLA_MB as shown in Figure 12, Figure 13 and Figure 15.
2. **Localization precision:** The FLPPL dynamic model presents superior precision results with the highest ratios in both WCL and WCWCL as indicated in Figure 16.
3. **Coverage:** In general, the static models perform better than the others in terms of network coverage. However, this not necessarily true when MA movement is constrained and limited. FLPPL consider three inputs in its movement decision, the RSSI signal, the distance between nodes and anchors and the number of neighbours of nodes which increases the number of localized nodes effectively. These results hold in both experiments when using different distances of movement as shown in Figure 17.

To conclude, we have shown that employing multiple inputs for forming a movement path has positive impacts in several regards.

## Figures and Tables

**Table 1:** General Comparison of Mobility-Assisted Movement schemes in WSNs.

Mobility Protocol	Localization Method	Localization Processing	Area	Anchor Type	Movement Path	Movement Constraints	Area Covered	Accuracy
SCAN	Range-free	Distributed	2-D	Mobile	Static	No	Yes	Low
Hilbert	Range-free	Distributed	2-D	Mobile	Static	No	Yes	Low
CIRCLES	Range-free	Distributed	2-D	Mobile	Static	No	No	Low
MAALRH	Range-free	Distributed	2-D	Mobile	Static	No	No	Low
Z-Curve	Range-free	Distributed	2-D	Mobile	Static	No	Yes	High
LMAT	Range-free	Distributed	2-D	Mobile	Static	No	Yes	High
H-Curves	Range-free	Distributed	2-D	Mobile	Static	No	Yes	High
DPMB	Range-free	Distributed	2-D	Mobile	Dynamic	No	No	Weak
NLA_MB	Range-free	Distributed	2-D	Mobile	Dynamic	Yes	Differs	High

**Table 2:** Input functions.

Input	Membership Function		
RSSI Level	Weak	Medium	Strong
Number of Neighbours	Low	Medium	High
Distance to each neighbour	Near	Medium	Far

**Table 3:** Node’s Neighbours Table.

N_Id	Type	Neighbours_#	Neighbours_Ids	Neighbours_Types	Neighbours_RSSI
20	0	3	48,147,226	0,0,0	−89.92,−57.91,−82.44

**Table 4:** The values of membership functions in FLPPL.

RSSI	Neighbours	Distance
Weak: [−102 −100 −90 −70]	Low: [−0.5 0 6 8]	Near: [−0.25 0 3.125 6.25]
Medium: [−90 −70 −30]	Medium: [6 8 12]	Medium: [3.125 6.25 9.375]
Strong: [−70 0 2]	High: [8 12 25 25.5]	Far: [6.25 9.375 12.5 12.75]

**Table 5:** Output functions.

Output	Membership Functions
	Very Weak
	Weak
	Little Weak
	Little Medium
Chance	Medium
	High Medium
	Little Strong
	Strong
	Very Strong

**Table 6:** The Fuzzy rules of if-then.

RSSI	Neighbours	Distance	Chance
Weak	Low	Far	Very Weak
Weak	Low	Medium	Weak
Weak	Low	Near	Little Weak
Weak	Medium	Far	Weak
Weak	Medium	Medium	Little Weak
Weak	Medium	Near	Little Medium
Weak	High	Far	Little Weak
Weak	High	Medium	Little Medium
Weak	High	Near	Medium
Medium	Low	Far	Little Weak
Medium	Low	Medium	Little Medium
Medium	Low	Near	Medium
Medium	Medium	Far	Little Medium
Medium	Medium	Medium	Medium
Medium	Medium	Near	High Medium
Medium	High	Far	Medium
Medium	High	Medium	High Medium
Medium	High	Near	Little Strong
Strong	Low	Far	Medium
Strong	Low	Medium	High Medium
Strong	Low	Near	Little Strong
Strong	Medium	Far	High Medium
Strong	Medium	Medium	Little Strong
Strong	Medium	Near	Strong
Strong	High	Far	Little Strong
Strong	High	Medium	Strong
Strong	High	Near	Very Strong

**Table 7:** Simulation values and parameters.

Parameters	Symbol	Value
Network size (*m*)	*S*	100 × 100
Number of mobile anchors	*M*	1
Number of unknown nodes	*N*	250
Maximum movement distance (*m*)	$d_{max}$	35, 70, 105, 140, 175
Path loss exponent	β	3.5
Power loss (dB) at $d_{0}$	PL(d0)	−60
Reference point (*m*)	d0	1
Standard deviation of noise	σ	3, 5, 7, 9
Simulation run		50

## Abbreviations

The following abbreviations are used in this manuscript:

BRF	Breadth-First
BTG	Backtracking Greedy
CH	Cluster Head
DPMB	Dynamic Path of Mobile Beacon algorithm
FIS	Fuzzy Logic Inference System
FL	Fuzzy Logic
FLC	Fuzzy Logic Controller
FLCFP	Fuzzy Logic Cluster Formation Protocol
FLPPL	Fuzzy-Logic based Path Planning for mobile anchor-assisted Localization
GPS	Global Positioning System
LMAT	Localization algorithm with a Mobile Anchor node based on Trilateration
MA	Mobile Anchor
MAALRH	Mobile Anchor-Assisted Localization algorithm based on a Regular Hexagon
NLA_MB	Node Localization Algorithm with Mobile Beacon node
PSO	Particle Swarm Optimization
RN	Reference Node
RSSI	Received Signal Strength Indicator
WCL	Weighted Centroid Localization algorithm
WCWCL	Weight-Compensated Weighted Centroid Localization
WSN	Wireless Sensor Network
UAV	Unmanned Aerial Vehicle
UN	Unknown Node
